# Retraction: Mechanism of action of Asparagus officinalis extract against multiple myeloma using bioinformatics tools, *in silico* and *in vitro* study

**DOI:** 10.3389/fphar.2025.1563699

**Published:** 2025-01-27

**Authors:** Frontiers Editorial Office

The Journal retracts the 09 May 2023 article cited above.

Following publication, concerns were raised regarding integrity of the images in the published figures. Image duplication was identified in Figure 5C and 6A. In addition, concerns were raised regarding scientific validity of the article. An investigation was conducted in accordance with Frontiers’ policies. The authors failed to provide a satisfactory explanation and as a result, the conclusions of the article have been deemed unreliable and the article has been retracted.

Further, the investigation confirmed that a non-genuine email address was used to impersonate Alaa El-Din Bekhit and the real Alaa El-Din Bekhit did not take any actions on this manuscript.

The author(s) does not agree to this Retraction.

This retraction was approved by the Chief Editors of Frontiers in Pharmacology and the Chief Executive Editor of Frontiers.

